# Growing Importance of Natural Products Research

**DOI:** 10.3390/molecules25010006

**Published:** 2019-12-18

**Authors:** Pavel B. Drasar, Vladimir A. Khripach

**Affiliations:** 1 Department of Chemistry of Natural Compounds, University of Chemistry and Technology, Technicka 5, 166 28 Prague, Czech Republic; 2 Institute of Bioorganic Chemistry, National Academy of Sciences of Belarus, 5/2 Academician V. F. Kuprevich Street, BY-220141 Minsk, Belarus; khripach@iboch.bas-net.by

Natural products and preparations based on them play a stable and ever-increasing role in human and veterinary medicine, agriculture, in food and the cosmetic industry, and in other increasing numbers of fields. Their importance is based on the fact that they are mostly bound to renewable sources, which in fact makes them valuable within a circular economy, inter alia. At the same time, natural products give the origin of stereochemistry, optical activity, regioselectivity, chirality, and many other concepts and directions within science, development, and industry in a scope, which is indispensable. They serve as a constant powerful stimulus and model that inspires researchers to create new effective tools, similar to natural ones for controlling bioregulation mechanisms and solving practical problems. This was the reason for organizing this Special Issue aimed to underline current developments in all fields connected to natural products.

Hence, the Molecules Special Issue “Synthesis, Study and Utilization of Natural Products” brought in 15 papers, four reviews, and 11 full research communications.

The scope of the selected topics was rather broad, it showed the importance of the pegylated purpurin 18 for photodynamic therapy of cancer [1], it presented the anti-platelet aggregation activity study of ginkgolide-1,2,3-triazole derivatives [2], it showed that the overexpression of the melatonin synthesis-related gene *SLCOMT1* improves the resistance of tomato to salt stress [3]. Another study revealed the effects of isosorbide incorporation into flexible polyurethane foams: reversible urethane linkages and antioxidant activity [4]. The synthesis and in vitro evaluation of caffeoylquinic acid derivatives as potential hypolipidemic agents [5] and the first total synthesis of varioxiranol A [6] were also presented. Another study introduced to the readers the preparation of polysaccharides from *Ramulus mori*, and their antioxidant, anti-inflammatory, and antibacterial activities [7]. Studied were also the effect of enzymolysis on the performance of soy protein-based adhesive [8] and the study of new octadecanoid enantiomers from the whole plants of *Plantago depressa* [9]. Connected studies that combined the biological properties of another type of secondary metabolite described the biosynthesis of fluorescent β subunits of C-phycocyanin from *Spirulina subsalsa* in *Escherichia coli*, and their antioxidant properties [10] and presented the synthesis of the sex pheromone of the tea tussock moth based on a resource chemistry strategy [11].

Review articles described well the synthesis and anticancer activity of CDDO and CDDO-Me, two derivatives of natural triterpenoids [12], the advances in biosynthesis, pharmacology, and pharmacokinetics of pinocembrin, a promising natural small-molecule drug [13], as well as recent advances in the discovery and biosynthetic study of eukaryotic RiPP natural products [14]. Another review article addressed the issue of whether polyphenols could help in the control of rheumatoid arthritis [15].

Summing up, the current development in the chemistry of natural products proved to be so exciting that now Molecules itself organized recently several special issues oriented to this unfinished and fruitful field of the activity of the world chemical community. It is important to wish chemists and their friends in connected fields much enthusiasm and success in their work as it brings so many useful fruits and tools for all humankind.
